# Supplementary material for: Enabling three-dimensional porous architectures via carbonyl functionalization and molecular-specific organic-SERS platforms
Source: Nat Commun. 2021 Oct 21;12:6119. doi: 10.1038/s41467-021-26385-7 (PMC8531383; doi:10.1038/s41467-021-26385-7)
Supplement: Supplementary file 1 — Supplementary Information [file 41467_2021_26385_MOESM1_ESM.pdf]

Supplementary Information for

Enabling three-dimensional porous architectures via carbonyl functionalization and molecular specific organic-SERS platforms

Ibrahim Deneme[1],[†] Gorkem Liman[2],[†] Ayse Can[1], Gokhan Demirel[2]*, Hakan Usta[1]*

[1] Department of Nanotechnology Engineering, Abdullah Gül University, 38080 Kayseri, Turkey.

[2] Bio-inspired Materials Research Laboratory (BIMREL), Department of Chemistry, Gazi University, 06500 Ankara, Turkey.

[†] These authors contributed equally to this article.

***Correspondence to:**

Prof. Hakan Usta (E-mail: hakan.usta@agu.edu.tr)

Prof. Gokhan Demirel (E-mail: nanobiotechnology@gmail.com)

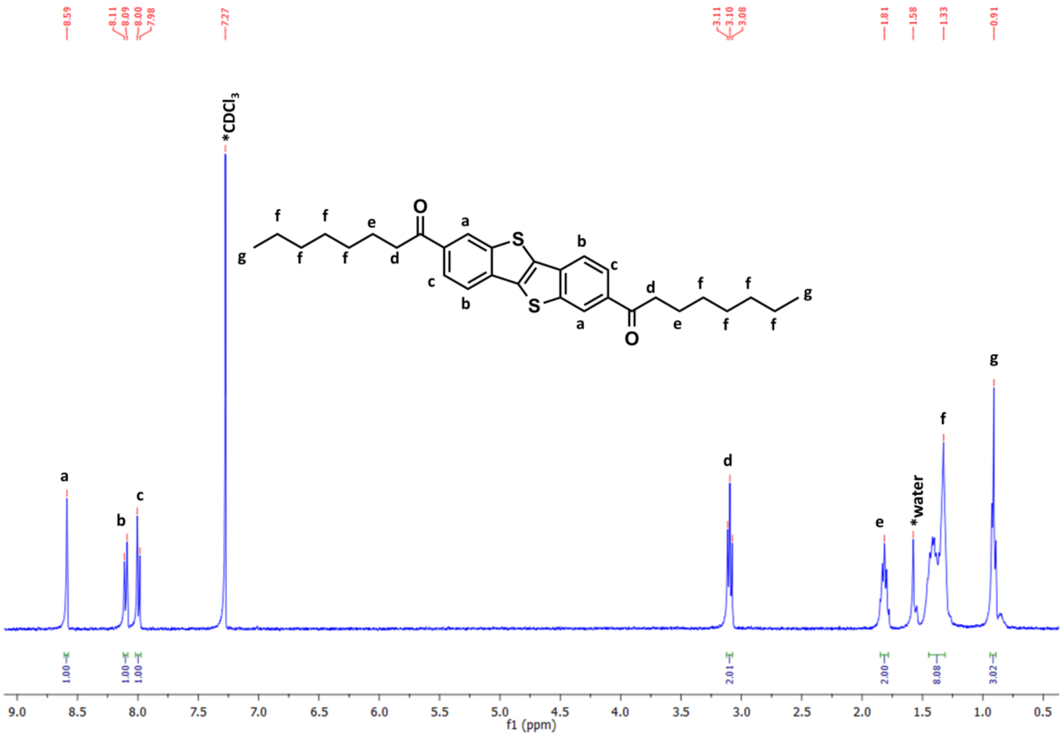

Supplementary Figure 1. ^{1}H NMR spectra of **D(C$_7$CO)-BTBT** measured in CDCl$_3$.

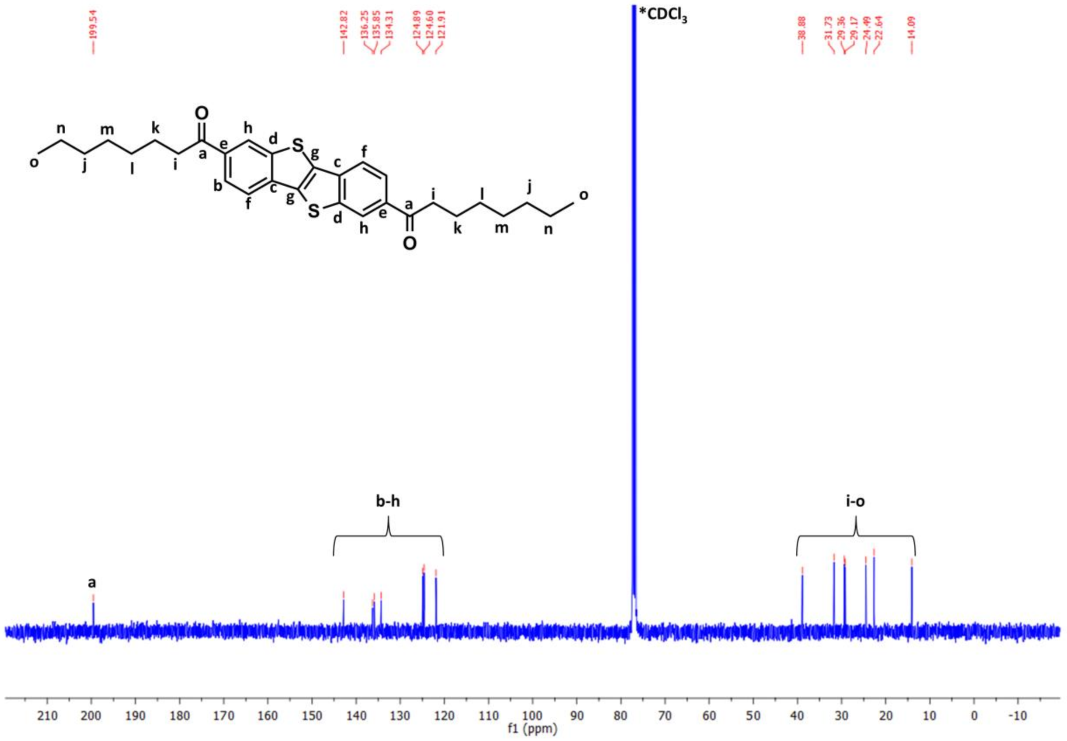

Supplementary Figure 2. ^{13}C NMR spectra of **D(C$_7$CO)-BTBT** measured in CDCl$_3$.

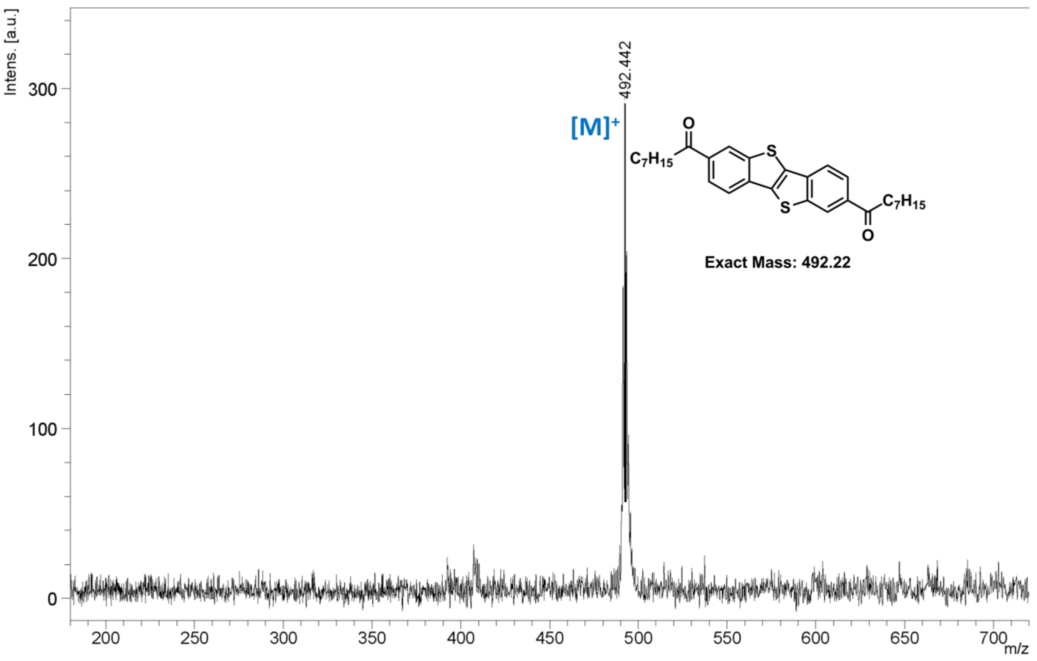

Supplementary Figure 3. Positive ion and linear mode MALDI TOF-MS spectrum of **D(C_7CO)-BTBT**.

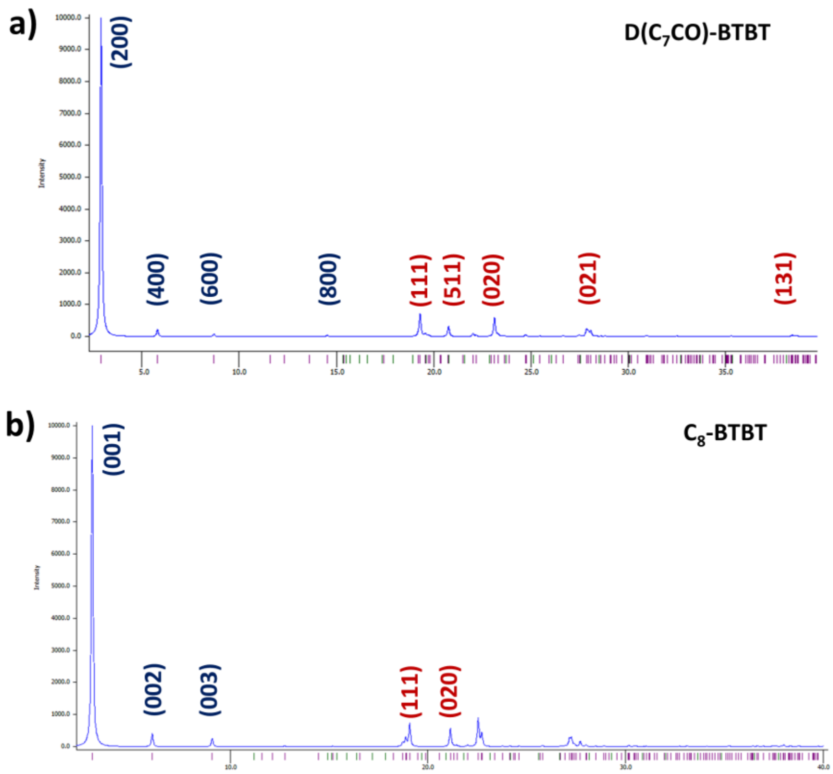

Supplementary Figure 4. Simulated XRD powder patterns based on the single-crystal structures for **D(C_7CO)-BTBT** (a) and **C_8-BTBT** (b) indicating the selected matching diffraction peaks and lattice planes. The dark blue and red assignment colors refer to the edge-on and face-on molecular orientations, respectively, within that lattice plane with respect to the substrate plane.

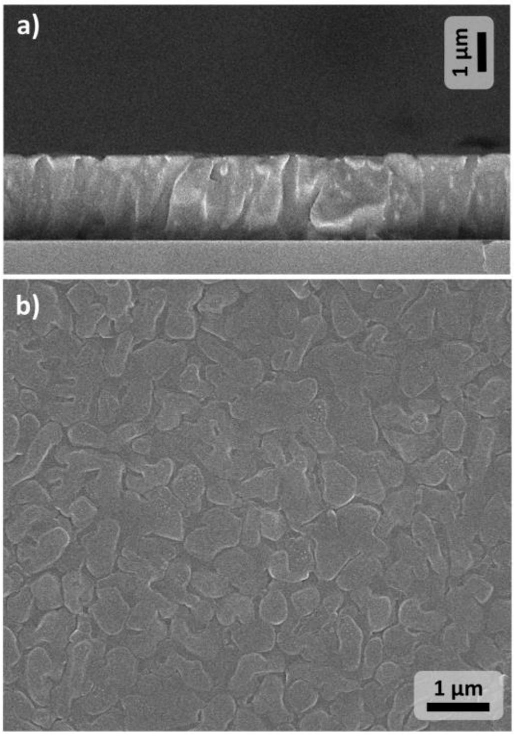

Supplementary Figure 5. Cross-sectional (a) and top-view (b) SEM images of the **BTBT** film deposited via physical vapor deposition method. Scale bars are shown separately for each SEM image.

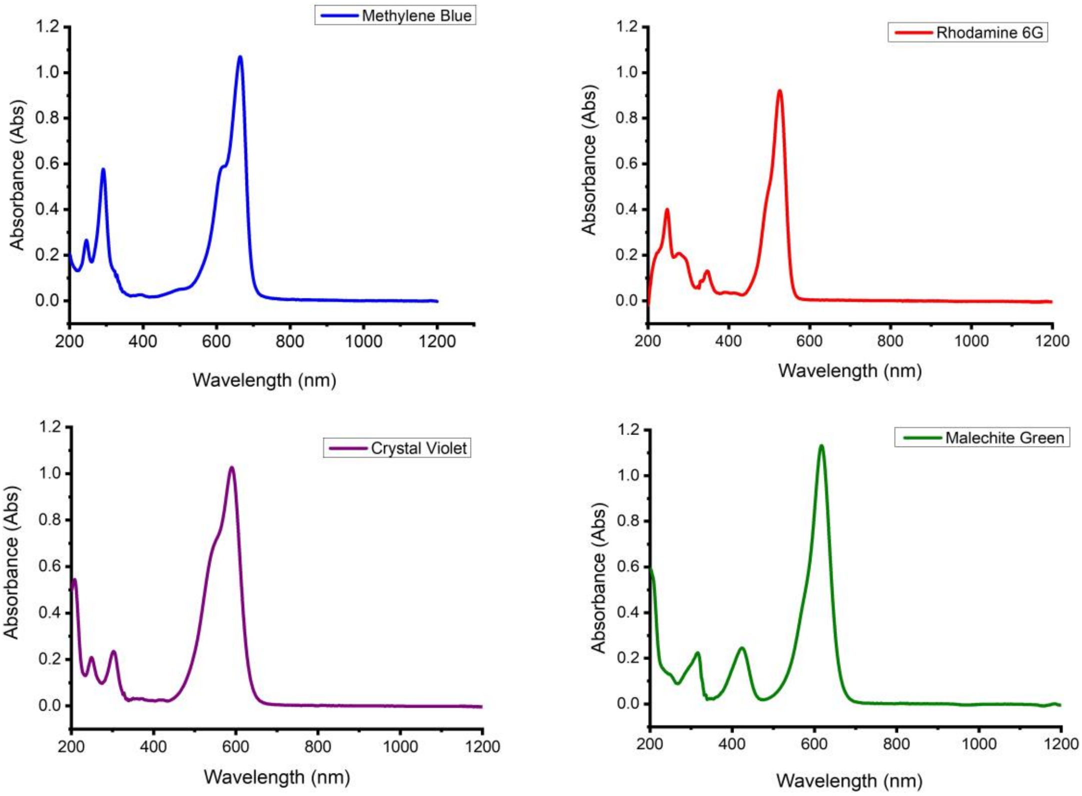

Supplementary Figure 6. UV-vis optical absorption spectra of **MB**, **R6G**, **CV**, and **MG**.

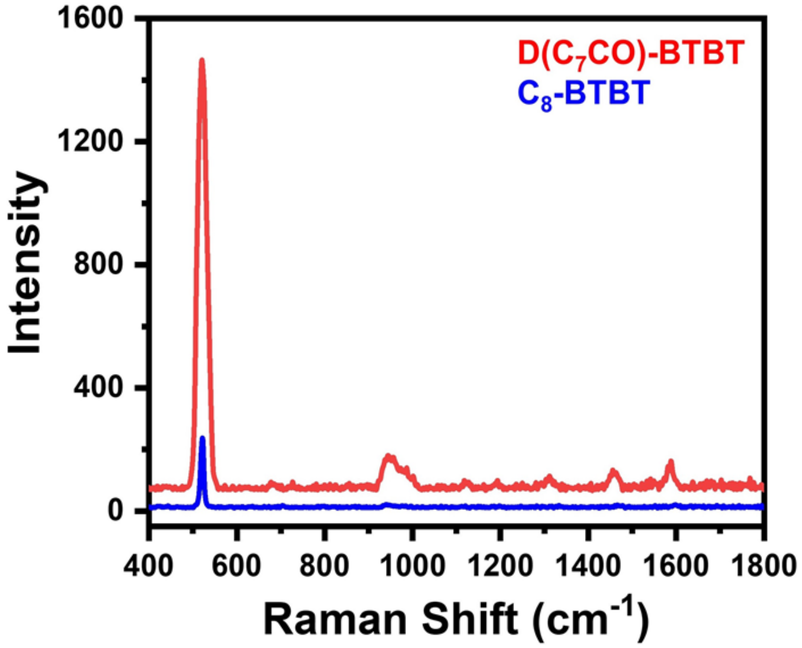

Supplementary Figure 7. Raman spectra for pristine **D(C_7CO)-BTBT** and **C_8-BTBT** films.

Supplementary Table 1. Band positions (in cm^{-1}) and their assignments for SERS spectra of **MB**, **R6G**, **CV**, and **MG** on **C_8-BTBT** and **$D(C_7CO)$-BTBT** films.

MB on C_8-BTBT films (cm^{-1})	MB on $D(C_7CO)$-BTBT films (cm^{-1})	Band Assignments[1,2]
443	445	δ(C-N-C)
763	766	γ(C-H)
1174	1181	ν(C-N)
1382	1391	α(C-H)
1432	1435	ν(C-N)
1614	1621	$\nu(C\text{-}C)_{ring}$

ν, stretching; α, in-plane ring deformation; γ, out-of-plane bending; and δ, skeletal deformation.

R6G on C_8-BTBT films (cm^{-1})	R6G on $D(C_7CO)$-BTBT films (cm^{-1})	Band Assignments[3,4]
-	612	$\beta(C─C)_{xanthene\ ring}/\beta(C─C)_{phenyl\ ring}$
-	774	γ(C─H)
-	1125	$\beta(C─H)_{xanthene\ ring}/\beta(C─H)_{phenyl\ ring}$
-	1186	$\beta(C─H)_{xanthene\ ring}$
-	1314	Hybrid mode (xanthene, phenyl ring and NHC_2H_5)
-	1651, 1512, 1360	$\nu_s(C─C)_{xanthene\ ring}$

ν, stretching; β, in-plane bending; and γ, out-of-plane bending.

CV on C_8-BTBT films (cm^{-1})	CV on $D(C_7CO)$-BTBT films (cm^{-1})	Band Assignments[5,6]
-	424	$\delta(C─N─C)/\delta(C─C_{center}─ C)$
-	726	ν(C─N)
-	916, 940	$\delta(C─C_{center}─C)$
-	1178	$\nu_{as}(C─C_{center}─C)$
-	1392	$\delta(CH_3)/\alpha(C─H)/\delta(C─C─C)_{ring}$
-	1540, 1585	$\nu_s(C_{ring}─N)/\delta_s(CH_3$
-	1622	ν_s(C─C)

ν, stretching; α, in-plane ring deformation; γ, out-of-plane bending; and δ, skeletal deformation.

MG on C_8-BTBT films (cm^{-1})	MG on $D(C_7CO)$-BTBT films (cm^{-1})	Band Assignments[7,8]
-	436	γ(Ph─C─Ph)
-	799	$\gamma(C─H)_{ring}$
-	919	Ring skeletal vibration
-	1179	$\beta(C─H)_{ring}$
-	1223	$\delta_s(C─C)_{ring}$
-	1299	$\nu_s(C─C)_{ring}$
-	1367, 1398	ν_s(N─Ph─C)
-	1591, 1619	ν_s(C─C)

ν, stretching; β, in-plane bending; γ, out-of-plane bending; and δ, skeletal deformation.

Supplementary References

1. Xiao, G.N., & Man, S.Q. Surface-enhanced Raman scattering of methylene blue adsorbed on cap-shaped silver nanoparticles. *Chem. Phys. Lett.* **447**, 305-309 (2007).
2. Naujok, R.R., Duevel, R.V. & Corn, R.M. Fluorescence and Fourier Transform surface-enhanced Raman scattering measurements of methylene blue adsorbed onto a sulfur-modified gold electrode. *Langmuir* **9**, 1771-1774 (1993).
3. Wu, C., Chen, E. & Wei, J. Surface enhanced Raman spectroscopy of Rhodamine 6G on agglomerates of different-sized silver truncated nanotriangles. *Colloids Surf. A Physicochem. Eng. Asp*. **506**, 450-456 (2016).
4. Xu, W., Ling, X., Xiao, J., Dresselhaus, M.S., Kong, J., Xu, H., Liu, Z. & Zhang, J. Surface enhanced Raman spectroscopy on a flat graphene surface. *Proc. Natl. Acad. Sci. U. S. A.* **109**, 9281-9286 (2012).
5. Cañamares, M.V., Chenal, C., Birke, R.L. & Lombardi, J.R. DFT, SERS, and single-molecule SERS of crystal violet. *J. Phys. Chem. C* **112**, 20295-20300 (2008).
6. Morovvati, B. & Malekfar, R. Surface enhanced Raman scattering of crystal violet with low concentrations using self-assembled silver and gold-silver core-shell nanoparticles. *Int. J. Opt. Photonics* **13**, 89-96 (2019).
7. He, L., Kim, N.J., Li, H., Hu, Z. & Lin, M. Use of a fractal-like gold nanostructure in surface-enhanced Raman spectroscopy for detection of selected food contaminants. *J. Agric. Food Chem*. **56**, 9843-9847 (2008).
8. Cen, Q., He, Y., Xu, M., Wang, J. & Wang, Z. Wavelength dependent resonance Raman band intensity of broadband stimulated Raman spectroscopy of malachite green in ethanol. *J. Chem. Phys*. **142**, 114201 (2015).
